# Effects of the Different Solid Deposits on the Corrosion Behavior of Pure Fe in Water Vapor at 500°C

**DOI:** 10.1155/2020/6280725

**Published:** 2020-09-11

**Authors:** Yanbing Tang, Xinwang Shen, Zhihong Liu, Ying Li

**Affiliations:** ^1^Institute of Metal Research, Chinese Academy of Sciences, 110016 Shenyang, China; ^2^Marine Equipment and Technology Institute, Jiangsu University of Science and Technology, 212003 Zhenjiang, China; ^3^School of Materials Science and Technology, Jiangsu University of Science and Technology, 212003 Zhenjiang, China

## Abstract

A comprehensive corrosion investigation of pure Fe in an environment of solid sodium salt deposit (i.e., NaCl or Na_2_SO_4_) with mixtures of H_2_O and O_2_ at 500°C was conducted by mass gain measurement, X-ray diffraction (XRD), scanning electron microscope (SEM), potentiodynamic polarization, and electrochemical impedance spectroscopy (EIS). The results showed that corrosion rates were accelerated with solid NaCl or Na_2_SO_4_ deposit due to their reaction with the formed protective scale of Fe_2_O_3_ and subsequently resulted in its breakdown. The corrosion rate of pure Fe with solid NaCl is higher than that with solid Na_2_SO_4_ because of the lower activation energy (*E*_a_) for chemical reaction of Fe in solid NaCl+H_2_O+O_2_ (i.e., 140.5 kJ/mol) than that in solid Na_2_SO_4_+H_2_O+O_2_ (i.e., 200.9 kJ/mol). Notably, the electrochemical corrosion rate of pure Fe with solid NaCl deposit, 1.16 × 10^−4^ A/cm^2^, was a little lower than that with solid Na_2_SO_4_ deposit.

## 1. Introduction

Corrosion of metal materials is severe in the environment with solid salt deposit and dry or wet O_2_ at medium and high temperatures [1–18], especially for turbine blades in planes or ships and power boilers. Due to their excellent mechanical properties, low cost, and ease of machining, pure Fe and its alloys are the popular materials that were investigated in solid alkali chloride deposit in dry or wet O_2_ [1–7]. Most researchers [2, 5, 6] thought that the solid NaCl could react with Fe_2_O_3_ to generate Cl_2_ via the reaction, 2NaCl + Fe_2_O_3_ + 1/2O_2_ = Na_2_Fe_2_O_4_ + Cl_2_. Then, Cl_2_ could react with Fe to form FeCl(s), i.e., Cl_2_ + Fe = FeCl_2_(s). As such, FeCl_2_(s) would continuously evaporate under low vapor pressure at high temperature. The FeCl_2_ vapor would diffuse outward through cracks and pores of the scale. Finally, the FeCl_2_ vapor would react with O_2_ to form Fe_3_O_4_ and/or Fe_2_O_3_ when they met during the process. Apparently, the solid NaCl could react with the oxidation scale that formed in dry or wet air at medium and high temperatures and lead to the breakdown of the protective scale to accelerate the corrosion rate of materials. Folkenson et al. [7] proposed a new mechanism as follows. Solid KCl reacts with O_2_ and H_2_O to generate chloride ions (i.e., 2KCl + 1/2O_2_ + H_2_O + 2e^−^⟶2KOH (ads) + 2Cl^−^(ads)) and subsequently react with iron ions to form FeCl_2_(s). Cao et al. [3] postulated a hypothesis of “dynamic water film” in which H_2_O molecules were continuously being absorbed on and evaporated from the surface of the material. The electrochemical corrosion might occur in the dynamic water film, which accelerates the corrosion of metal [3]. Shu et al. [10] used impedance spectroscopy to investigate the corrosion mechanisms of pure Fe and pure Cr with solid NaCl deposit in water vapor at 600°C. According to the analysis of the resistance and capacitance of corrosion scale, the electrochemical corrosion was proved to occur in the corrosion environments. After that, Tang et al. [1] investigated the interaction between chemical reactions and electrochemical reactions of pure Fe in this corrosion environment. The chemical reactions and electrochemical reactions follow “ce mechanism,” in which Fe and Fe_2_O_3_ first react chemically with NaCl, water vapor, and oxygen to generate HCl (g). Then, the HCl (g) reacts with pure Fe electrochemically via a one-electron electrochemical reduction to form H_2_.

Compared to the alkali chlorides, the corrosion mechanisms of metal/alloys with solid sulfate are lacking. However, the corrosion mechanisms are focused on molten sulfate. Recently, many studies have been carried out on the corrosion behavior of metals/alloys in a molten Na_2_SO_4_ environment [19–25] and many corrosion mechanisms have been proposed. One of the well-known mechanisms is the sulfidation model [19, 20], in which the formation of sulfides accelerates the corrosion. The other one is the acidic-basic fluxing [19, 21–24] mechanism, in which dissolution of the protective oxide scales, due to formation of basic Na_2_O, was considered the reason for the accelerated corrosion. Moreover, based on the electrochemical mechanism [25], corrosion was considered an electrochemical reaction in which the transfer of electrons accelerated the corrosion. Tang et al. [26] investigated the corrosion behavior of pure Fe under solid Na_2_SO_4_ deposit in wet oxygen flow at 500°C. The results showed that the corrosion of Fe includes chemical corrosion and electrochemical corrosion. The chemical reaction and electrochemical reaction follows the “ce mechanism.” Fe and Fe_2_O_3_ first react chemically with Na_2_SO_4_, water vapor, and oxygen to generate H_2_SO_4_ (g). And then, the H_2_SO_4_ (g) reacts with pure Fe electrochemically via a one-electron electrochemical reduction to form H_2_. The coeffect between deposited solid Na_2_SO_4_ and H_2_O+O_2_ certainly exists and significantly accelerates the corrosion of pure Fe.

NaCl and Na_2_SO_4_ are normal corrosive mediums. The corrosion of materials in solid salt environment depends on the anion of the salt [18, 27]. However, the effects of the different solid deposits (NaCl and Na_2_SO_4_) on the corrosion behavior of pure Fe in water vapor are still unclear. In this paper, the corrosion differences of pure Fe with solid NaCl and solid Na_2_SO_4_ deposit in water vapor were comparatively studied to cognize the corrosion behaviors of materials in the corrosion profoundly.

## 2. Experimental

**Figure 1:**
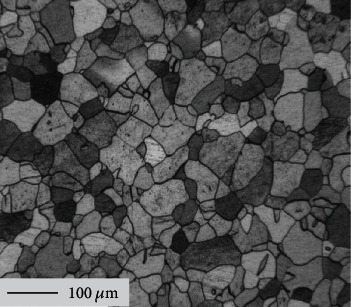
Metallograph of pure Fe.

The pure Fe (99.9%) was used as experimental specimen. The metallography of the specimen is shown in Figure 1. The microstructure of pure Fe is ferrite. The maximum grain size is about 100 *μ*m. Before the experiment, the sample was ground using silicon-carbide abrasive papers down to 1000 grit, degreased in acetone then ethanol, and dried in air before use. The NaCl and Na_2_SO_4_ are of analytical purity (≥99.5%). The solid salt was deposited on the preheated Fe sample surface by repeatedly brushing and drying a salt-saturated solution. The mass of salt was about 4 mg/cm^2^. The temperature of the furnace was controlled at 500°C. H_2_O came from an 80°C water bath. Pure O_2_ was passed through the glass bubbler with a flux of 200 ml/min.

The corrosion test was carried out in a thermal balance [2]. To prevent the H_2_O from condensing in the upper part of the thermal balance, a counterflow of N_2_ was passed through the apparatus at 150 ml/min. After the furnace was heated to the desired temperature and the gas flow was stabilized, the specimen was quickly suspended into the furnace tube, and the test was started. All the measurements were carried out at ambient pressure. After the tests, the specimens were further examined by XRD and SEM.

A special three-electrode system was built for the electrochemical measurements in this particular environment [1]. To decrease the resistance of the solution and obtain a uniform electric field, the reference electrodes consisted of four platinum wires, each with a diameter 0.4 mm, and the counter electrode was a circular strip of platinum foil about 2 mm wide. All potential values in this paper were reported versus this platinum reference electrode. The Fe working electrode was a rod 10 mm long and 5 mm diameter. The three electrodes were placed in quartz tubes, which acted as insulators. All the gaps were sealed by high-temperature inorganic glue. The three-electrode system after solid NaCl and solid Na_2_SO_4_ deposition was directly put into the furnace at the desired temperature and with water vapor for electrochemical measurements.

A PAR2273 Electrochemical Measurement System manufactured by EG&G was used for all electrochemical measurements, which also has the function to compensate the resistance between reference electrode and working electrode. In the galvanic corrosion measurement, the ratio of anodic area to cathodic area is 1 : 2. In the potentiodynamic polarization measurements, the measurements were carried out after 1000 s in the corrosion environment for obtaining an electrochemical stability and the scan rate was 1 mV/s. The resistance between reference and working electrodes was compensated during measurements according to the design of electrochemical system and testing work station. All measurements were repeated more than three times.

## 3. Results and Discussion

**Figure 2:**
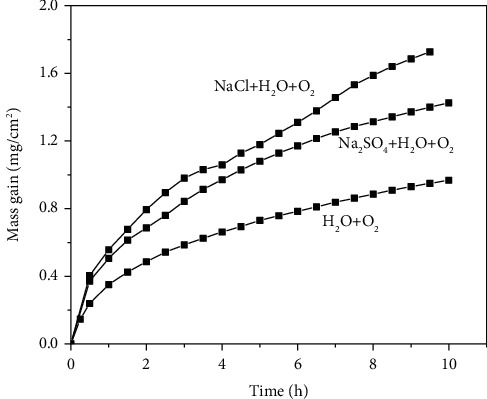
Mass gain plots of pure Fe with and without solid NaCl or Na_2_SO_4_ deposit in O_2_ containing water vapor at 500°C.

Figure 2 shows the mass gain of pure Fe as a function of time at 500°C with and without solid NaCl or Na_2_SO_4_ [26] in O_2_ containing water vapor. As is seen from Figure 2, the corrosion of pure Fe is accelerated with solid NaCl or Na_2_SO_4_ deposit. Compared to the case with solid Na_2_SO_4_ deposit, the corrosion rate of pure Fe with solid NaCl deposit is slightly higher at all-time duration.

In our previous studies [1, 13], it is found that the corrosion of pure Fe in both corrosion environments includes a chemical corrosion process and an electrochemical corrosion process, while the overall corrosion is dominated by the chemical corrosion process with a percentage of over 90%. Herein, we investigated the differences of chemical corrosion that is influenced by NaCl and Na_2_SO_4_.

To affect the chemical corrosion rate, there are two aspects: (a) the protection of scale on the surface of pure Fe. The compact and integrated scale can restrain the corrosion of substrate. (b) The activity of corrosion reactants. As it is known, the corrosion rate would increase with a decreasing active energy. The details of the effects are discussed as follows.

**Figure 3:**
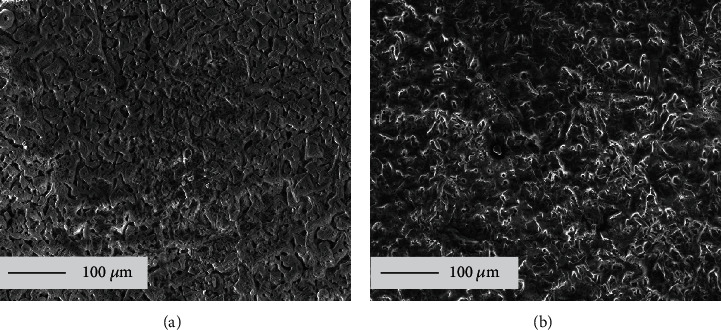
Surface morphologies of solid salt scale on pure Fe before experiment: (a) solid NaCl; (b) solid Na_2_SO_4_.

The scale includes solid salt deposition scale (NaCl or Na_2_SO_4_) and corrosion scale on the surface of pure Fe. Figures 3(a) and 3(b) show the surface morphologies of solid NaCl and Na_2_SO_4_, respectively, before corrosion test. The results showed that both salt scales are loose and porous. However, the solid NaCl film was much looser and more porous than solid Na_2_SO_4_ film, which led to an easy transport of H_2_O and O_2_ to the interface of pure Fe and solid NaCl film, promoting the chemical corrosion process of pure Fe.

**Figure 4:**
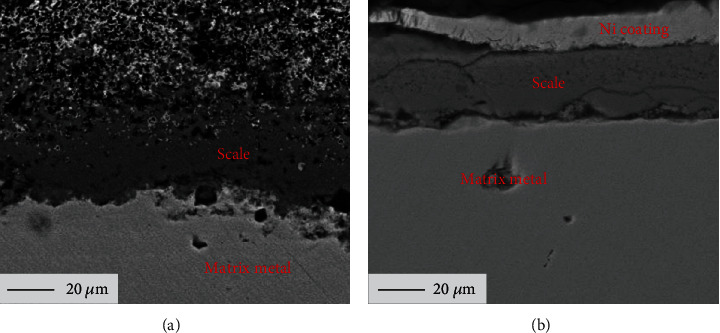
Cross-sectional morphologies of pure Fe with NaCl or Na_2_SO_4_ deposit in O_2_ flow with water vapor for 10 h: (a) solid NaCl and (b) solid Na_2_SO_4_.

The corrosion of materials with solid salt deposit in water vapor is different with that in aqueous solution. The corrosion scale would stay on the surface of substrate, which should restrain the corrosion of substrate.  Figure 4 shows the cross-sectional morphologies of pure Fe after 10 h corrosion at 500°C in NaCl + H_2_O + O_2_ (Figure 4(a)) and Na_2_SO_4_ + H_2_O + O_2_ (Figure 4(b)) [26]. It indicated that the corrosion scale formed on the surface of pure Fe was loose and porous in both corrosion environments. A number of volatile species are formed in the corrosion process, which could contribute to the formation of the loose and porous corrosion scale [6]. As a matter of fact, some green deposits were observed on the tube inner surface of the furnace after many hours of experiments, confirming the formation of volatile species. However, the scale formed in solid NaCl + H_2_O + O_2_ is looser and higher porosity than that formed in solid Na_2_SO_4_ + H_2_O + O_2_ [26]. This indicated that the reactants (H_2_O and O_2_) could be easier to transport through the corrosion scale formed in the environment with NaCl. Eventually, it promotes the chemical corrosion process of pure Fe.

**Figure 5:**
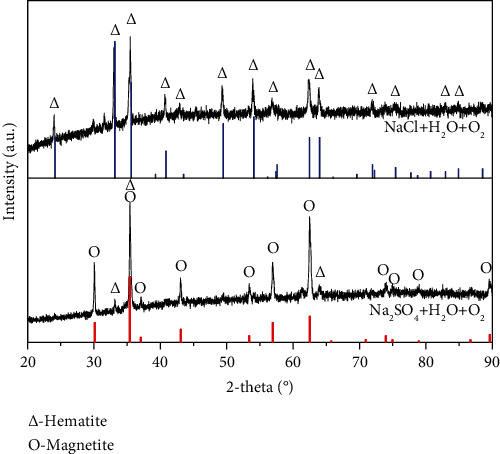
XRD results of the corrosion scale of pure Fe in water vapor with solid NaCl or Na_2_SO_4_ deposits at 500°C.

The components of the corrosion scale formed on the surface of pure Fe in solid NaCl + H_2_O + O_2_ or Na_2_SO_4_ + H_2_O + O_2_ [26] after 10 h corrosion are shown in Figure 5. The components of the scales in the two corrosion environments are remarkably different. The component of the scale on the surface of pure Fe formed in solid NaCl + H_2_O + O_2_ is hematite that mainly consists of Fe_2_O_3_, while the component of the scale on the surface of pure Fe formed in solid Na_2_SO_4_ + H_2_O + O_2_ mainly consists of Fe_3_O_4_ with a little of Fe_2_O_3_. According to the published research [27], the generation of Fe_2_O_3_ or Fe_3_O_4_ is closely relative to oxygen pressure. Fe_2_O_3_ would be generated at a relatively high oxygen pressure, while Fe_3_O_4_ would be generated at a relatively low oxygen pressure. From Figures 3 and 4, the NaCl scale is looser with higher porosity than Na_2_SO_4_ scale; meanwhile, the corrosion scale formed in the case of NaCl + H_2_O + O_2_ was also looser with higher porosity than that formed in the case of Na_2_SO_4_ + H_2_O + O_2_. The oxygen could transport inward through the corrosion scale and solid NaCl scale easily. The oxygen pressure in the corrosion scale that formed in NaCl + H_2_O + O_2_ is higher than that formed in Na_2_SO_4_ + H_2_O + O_2_. This is the reason why the components of the corrosion scales in the two corrosion environments were different.

The corrosion mechanism of pure Fe in the two corrosion environments could be understood on the basis of the components, morphologies of the corrosion scales, and published research.

For the case of solid NaCl, firstly, NaCl reacts with Fe_2_O_3_ and H_2_O to generate Na_2_Fe_2_O_4_ and HCl [4].

(1) $$\begin{array}{c} 2\text{NaCl}\left(\text{s}\right)+{\text{Fe}}_{2}{\text{O}}_{3}+{\text{H}}_{2}\text{O}={\text{Na}}_{2}{\text{Fe}}_{2}{\text{O}}_{4}+\text{HCl}. \end{array}$$

The generated HCl could react with Fe to form FeCl_2_ [4], meanwhile, HCl could also react with O_2_ to form Cl_2_ [27].

(2) $$\begin{array}{c} 2\text{HCl}+\text{Fe}=\text{FeC}{\text{l}}_{2}\left(\text{s}\right)+{\text{H}}_{2}\\ 4\text{HCl}+{\text{O}}_{2}=2\text{C}{\text{l}}_{2}+2{\text{H}}_{2}\text{O} \end{array}$$

The Cl_2_ could react with Fe to form FeCl_2_ [28].

(3) $$\begin{array}{c} \text{Fe}+\text{C}{\text{l}}_{2}=\text{FeC}{\text{l}}_{2}\left(\text{s}\right) \end{array}$$

The vapor pressure for FeCl_2_(s) is 4 × 10^−5^ Pa at 500°C. A continuous evaporation will take place [6].

(4) $$\begin{array}{c} \text{FeC}{\text{l}}_{2}\left(\text{s}\right)=\text{FeC}{\text{l}}_{2}\left(\text{g}\right) \end{array}$$

The FeCl_2_(g) diffuse outward through the scale and react with O_2_ and H_2_O to form a loose and porous Fe_2_O_3_ scale (see Figure 4(a)) [4].

(5) $$\begin{array}{c} 2\text{FeC}{\text{l}}_{2}+1/2\;{\text{O}}_{2}+2{\text{H}}_{2}\text{O}=\text{F}{\text{e}}_{2}{\text{O}}_{3}+4\text{HCl} \end{array}$$

For the case of solid Na_2_SO_4_, firstly, Na_2_SO_4_ reacts with Fe_2_O_3_ and H_2_O to generate Na_2_Fe_2_O_4_ and H_2_SO_4_ [26].

(6) $$\begin{array}{c} \text{N}{\text{a}}_{2}\text{S}{\text{O}}_{4}+\text{F}{\text{e}}_{2}{\text{O}}_{3}+{\text{H}}_{2}\text{O}=\text{N}{\text{a}}_{2}\text{F}{\text{e}}_{2}{\text{O}}_{4}+{\text{H}}_{2}\text{S}{\text{O}}_{4} \end{array}$$

The generated H_2_SO_4_ could react with Fe to form FeSO_4_.

(7) $$\begin{array}{c} {\text{H}}_{2}\text{S}{\text{O}}_{4}+\text{Fe}=\text{FeS}{\text{O}}_{4}+{\text{H}}_{2} \end{array}$$

According to the XRD results, the FeSO_4_ could react with O_2_ and H_2_O to form Fe_3_O_4_ and Fe_2_O_3_.

(8) $$\begin{array}{c} 5\text{FeS}{\text{O}}_{4}+5{\text{H}}_{2}\text{O}+{\text{O}}_{2}=\text{F}{\text{e}}_{3}{\text{O}}_{4}+\text{F}{\text{e}}_{2}{\text{O}}_{3}+5{\text{H}}_{2}\text{S}{\text{O}}_{4} \end{array}$$

The generation of H_2_SO_4_ and H_2_ led to the formation of many holes and cracks in the scale (see Figure 4(b)).

According to the morphologies shown in Figures 3 and 4, the more porous NaCl scale and corrosion scale formed in the NaCl + H_2_O + O_2_ promoted the chemical corrosion process. However, this did not fully explain why the corrosion rate of pure Fe in NaCl + H_2_O + O_2_ was higher than that in Na_2_SO_4_ + H_2_O + O_2_. The activation energy is a key parameter to estimate the chemical reaction rate. The lower the activation energy, the more atoms, ions, or molecules of substances are activated to transition state. Therefore, the rate of chemical reaction increases with decreases in chemical reaction activation energy.

**Figure 6:**
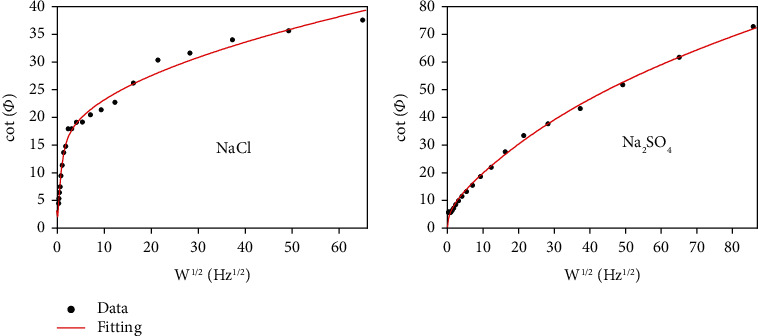
Frequency dependence of the phase angle of Fe in water vapor with NaCl or Na_2_SO_4_ deposits at 500°C at open circuit potential.

Both corrosion mechanisms of pure Fe in solid NaCl + H_2_O + O_2_ and solid Na_2_SO_4_ + H_2_O + O_2_ corrosion environments follow the ce mechanism [1, 26]. For the ce mechanism, the relationship between phase angle and frequency could be given as Equation (9) [28].

(9) $$\begin{array}{c} \cot\phi =\frac{\left({\left(2\omega \right)}^{1/2}/\lambda \right)+\left(1/\left(1+e^{i}\right)\right)\left\{\left(1/\left(1+K\right)\right)\right.{\left[\left({\left(1+g^{2}\right)}^{1/2}+g\right)/\left(1+g^{2}\right)\right]}^{1/2}+\left(K/\left(1+K\right)\right)+\left.e^{i}\right\}}{\left(1/\left(1+e^{i}\right)\right)\left\{\left(1/\left(1+K\right)\right)\right.{\left[\left({\left(1+g^{2}\right)}^{1/2}-g\right)/\left(1+g^{2}\right)\right]}^{1/2}+\left(K/\left(1+K\right)\right)+\left.e^{i}\right\}}, \end{array}$$

(10) $$\begin{array}{c} g=\frac{k_{1}+k_{2}}{\omega }, \end{array}$$

(11) $$\begin{array}{c} \lambda =\frac{k_{h}f}{D^{1/2}}\left(e^{-\alpha j}+e^{\beta j}\right), \end{array}$$

(12) $$\begin{array}{c} K=\frac{k_{1}}{k_{2}}, \end{array}$$

(13) $$\begin{array}{c} \beta =1-\alpha , \end{array}$$

(14) $$\begin{array}{c} j=\frac{nF}{RT}\left(E_{\text{d}.\text{c}.}-E_{1/2}^{r}\right), \end{array}$$

where *Φ* is used for representing for phase angle, *k*_1_ and *k*_2_ for chemical reaction rate constants, *ω* for angular frequency, *D* for diffusion coefficient, *k*_*h*_ for apparent heterogeneous rate constant, *f* for activity coefficient, *α* for charge transfer coefficient, *n* for number of electrons transferred, *E*_d.c._ for applied d. c. potential, *E*_1/2_^*r*^ for reversible half-wave potential, and *F*, *R*, and *T* for their conventional electrochemical meanings. The value of *k*_1_ can be calculated using Equation (9).  Figure 6 shows the relationship between frequency and phase angles of pure Fe in solid NaCl + H_2_O + O_2_ at 500°C. The plots have a maximum. It suggests that the corrosion mechanism of pure Fe in the two corrosion environments involves the interaction of the chemical and the electrochemical reactions, which is similar with pure Fe in solid Na_2_SO_4_ + H_2_O + O_2_ [26]. The calculated values of *k*_1_ for pure Fe in solid NaCl + H_2_O + O_2_ and solid Na_2_SO_4_ + H_2_O + O_2_ corrosion environments are 0.230 sec^−1^ and 0.031 sec^−1^, respectively. Therefore, the chemical corrosion rate of pure Fe in solid NaCl + H_2_O + O_2_ is higher than that in solid Na_2_SO_4_ + H_2_O + O_2_ because of its higher chemical reaction rate constant in the case with solid NaCl.

**Figure 7:**
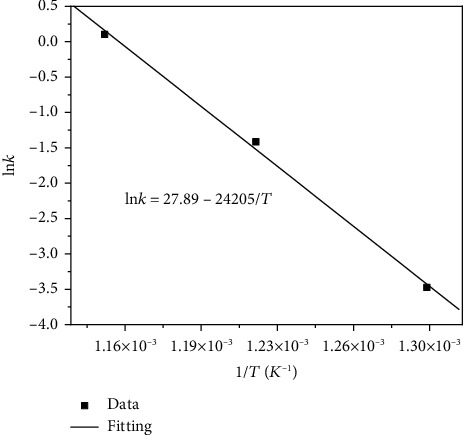
Temperature dependence of chemical reaction rate constant of pure Fe with solid Na_2_SO_4_ deposit in O_2_ flow with water vapor.

Chemical reaction rate is closely related with the activation energy. The lower the activation energy is, the higher the chemical reaction rate is. According to the logarithmic Arrhenius equation, the rate constant (*k*) dependence of temperature (*T*) is given by the relationship (*g*) [29]:

(15) $$\begin{array}{c} \ln\;k=\ln\;A-\frac{E_{\text{a}}}{RT}, \end{array}$$

where *k* is used for representing for the rate constant, *A* for a temperature-independent constant (often called the frequency factor), *T* for the absolute temperature, *R* for the universal gas constant, and *E*_a_ for the activation energy. According to Equation (15), a plot of ln*k* vs. 1/*T* gives a straight line with slope of *-E*_a_/*R*. The values of *E*_a_ for Fe in solid Na_2_SO_4_ + H_2_O + O_2_ corrosion environments can be obtained from the slope of Figures 7. The value is 200.9 kJ/mol. The value of *E*_a_ for Fe in solid NaCl + H_2_O + O_2_ corrosion environments is 140.5 kJ/mol [13]. The lower activation energy of Fe in solid NaCl + H_2_O + O_2_ accounts for its higher chemical reaction and the higher overall corrosion rate.

**Figure 8:**
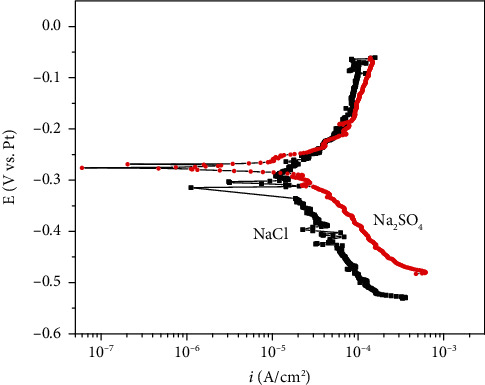
Potentiodynamic polarization plots of pure Fe in water vapor with solid NaCl or Na_2_SO_4_ deposits at 500°C.

The electrochemical corrosion of pure Fe in solid Na_2_SO_4_ + H_2_O + O_2_ corrosion environments has been shown in our earlier studies [1, 26]. The potentiodynamic polarization plot of pure Fe in solid NaCl + H_2_O + O_2_ at 500°C is shown in Figure 8. The anodic current densities of pure Fe in both two corrosion environments increase linearly with anodic potential increasing in the active polarization zone, which can be attributed to active dissolution in the aqueous environment, because the loose and porous corrosion scale could not inhibit the electrochemical corrosion. The cathodic reaction rate of pure Fe in solid Na_2_SO + H_2_O + O_2_ is higher than that in NaCl + H_2_O + O_2_. The electrochemical corrosion rates (*i*_corr_) were calculated by fitting the potentiodynamic polarization curves in the active polarization zones. The electrochemical corrosion current density (*i*_corr_) obtained was 1.16 × 10^−4^ A/cm^2^ and 1.30 × 10^−4^ A/cm^2^ for pure Fe in solid NaCl + H_2_O + O_2_ and solid Na_2_SO_4_ + H_2_O + O_2_ [26], respectively. The amount of Fe corroded by electrochemical reaction was obtained using Faraday's rule. After the calculation, the chemical reaction rates of pure Fe in the two corrosion environments within 1 h are 0.036 g/h/cm^2^ and 0.041 g/h/cm^2^, respectively. It must illustrate that the calculation time herein is in one hour, because the potentiodynamic polarization measurements were carried out within one hour, and there is no significantly variety of the electrochemical corrosion rate in one hour. This was proved by presented authors used an electrochemical instrument named CMB 1510B (based on weak polarization theory) manufactured by State Key Laboratory for Corrosion and Protection, to measure the electrochemical corrosion rate every 4 minutes during the whole corrosion reaction. The electrochemical corrosion rate of pure Fe in solid NaCl + H_2_O + O_2_ is slightly lower than that in solid Na_2_SO_4_ + H_2_O + O_2_.

As is well-known, charge transfer is the fundamental characteristic of the electrochemical reaction [30, 31]. The corrosion scale and solid salt scale are the key influence factors for electrochemical reaction rate. The corrosion scale of pure Fe formed in solid NaCl + H_2_O + O_2_ is looser and more porous than those formed in solid Na_2_SO_4_ + H_2_O + O_2_ (see Figure 4), and the solid NaCl scale is also looser and more porous than solid Na_2_SO_4_ scale (see Figure 3). The HCl could volatilize and diffuse through the looser and more porous corrosion scale and NaCl scale easily. Thus, the cathodic reaction rate of pure Fe in solid Na_2_SO_4_ + H_2_O + O_2_ is higher than that in solid NaCl + H_2_O + O_2_. As a consequence, the electrochemical corrosion rate of pure Fe in solid Na_2_SO_4_ + H_2_O + O_2_ is higher than that in solid NaCl + H_2_O + O_2_.

As a consequence, the electrochemical corrosion rate of pure Fe in solid Na_2_SO_4_ + H_2_O + O_2_ is higher than that of it in solid NaCl + H_2_O + O_2_. In addition, the components of the corrosion scales formed on the surface of pure Fe in solid NaCl + H_2_O + O_2_ and solid Na_2_SO_4_ + H_2_O + O_2_ after 10 h corrosion are shown in Figure 5. The component of the scales on the surface of pure Fe formed in solid NaCl + H_2_O + O_2_ is hematite that mainly consists of Fe_2_O_3_, while the component of the scales on the surface of pure Fe formed in solid Na_2_SO_4_ + H_2_O + O_2_ is Magnetite that mainly consists of Fe_3_O_4_. The protection of Fe_2_O_3_ is well than that of Fe_3_O_4_ [32], which also inhibit the electrochemical corrosion rate of pure Fe in solid NaCl + H_2_O + O_2_ corrosion environment.

## 4. Conclusion

The corrosion rate of the pure Fe is significantly accelerated under a NaCl or Na_2_SO_4_ deposit in an atmosphere of H_2_O + O_2_ at 500°C. Both the salts of NaCl and Na_2_SO_4_ could react with Fe_2_O_3_ to result in a breakdown of the protective scale and subsequently accelerate the corrosion rate of pure Fe.

Compared to the case in solid Na_2_SO_4_ + H_2_O + O_2_, the corrosion rate of pure Fe is much higher in solid NaCl + H_2_O + O_2_. The activation energy (*E*_a_) for chemical reaction of pure Fe in solid Na_2_SO_4_ + H_2_O + O_2_ is 200.9 kJ/mol, which is higher than that of pure Fe in solid NaCl + H_2_O + O_2_.

The percentage contribution of the electrochemical reactions in total corrosion is insignificant. It was also found that the electrochemical corrosion rate of pure Fe with solid NaCl deposit was 1.16 × 10^−4^ A/cm^2^, which was a little lower than that with solid Na_2_SO_4_ deposit.

## Data Availability

The data used to support the findings of this study are available from the corresponding author upon request.
